# Use of Radiation Therapy for Ataxia-Telangiectasia Mutated (ATM)-Mutation Metastatic Renal Cell Carcinoma: A Case Report

**DOI:** 10.7759/cureus.64781

**Published:** 2024-07-17

**Authors:** Steven N Seyedin, Garrett Harada, Eleen Garemanian, Desiree Rafizadeh, Dalia Kaakour, Sami Dwabe, Michael Daneshvar, Nataliya Mar

**Affiliations:** 1 Radiation Oncology, University of California San Francisco Medical Center, San Francisco, USA; 2 Radiation Oncology, University of California Irvine Medical Center, Orange, USA; 3 Biology, University of California Irvine, Irvine, USA; 4 Radiation Oncology, University of California Irvine School of Medicine, Irvine, USA; 5 Hematology and Oncology, University of California Irvine Medical Center, Orange, USA; 6 Urology, University of California Irvine Medical Center, Orange, USA

**Keywords:** radiation sensitizers, oligo metastatic disease, stereotactic ablative radiation, atm mutation, metastatic rcc

## Abstract

Papillary renal cell carcinoma (pRCC) is a rare kidney cancer with limited treatment options and poor outcomes when metastatic. We present a case of a 42-year-old male with metastatic pRCC harboring a somatic ataxia-telangiectasia mutated (ATM) mutation who was treated at our institution. After progression of disease (POD) on ipilimumab/nivolumab, followed by POD on cabozantinib, the patient was treated with radiation therapy to metastatic cervical lymphadenopathy to 60 Gy in 15 fractions as well as retroperitoneal lymphadenopathy to 36 Gy in 9 fractions, which was curtailed due to intolerance. This was followed by sequential systemic therapy with a poly (ADP-ribose) polymerase (PARP) inhibitor and pembrolizumab, which was also discontinued due to adverse effects. Despite not receiving any treatment for 10 months, his disease remains stable. We believe that the prolonged progression-free survival of this patient with ATM-mutation metastatic pRCC is likely due to the enhanced sensitivity of the tumor to radiation therapy due to ATM loss.

## Introduction

While renal cell carcinoma (RCC) is the seventh most common malignancy in the United States; papillary RCC (pRCC) represents nearly 8% of all newly diagnosed RCC [1,2]. Due to its rarity, no frontline systemic therapy regimen is preferred as large randomized controlled trials in the pRCC population are lacking. Current National Comprehensive Cancer Network (NCCN) guidelines list cabozantinib or clinical trial referral, as recommended options in previously untreated pRCC patients [3]. Other recommended regimens include combinations of an immune-oncology (IO) agent with a vascular endothelial growth factor (VEGF) tyrosine kinase inhibitor (TKI) or TKI with a mammalian target of rapamycin (mTOR) inhibitor as well as single-agent IO or TKIs. Unfortunately, progression-free survival (PFS) with these systemic therapy options remains under 12 months so additional therapeutic options are needed [4].

Studies examining the use of definitive radiotherapy for metastatic RCC have shown promising results, with excellent rates of local control of 85% at one year or higher [5-8]. Meanwhile, phase 2 studies demonstrated one-year PFS of over 50% post-radiation in the front-line metastatic or relapsed RCC setting [9,10]. However, the vast majority of patients in these studies had clear-cell RCC. The role of radiation therapy for pRCC remains poorly defined. Further, certain genomic alterations have previously been associated with improved responses to radiotherapy in solid tumors (or RCC), including mutations in DNA damage repair genes [11,12]. We report a case of metastatic pRCC with a somatic ataxia-telangiectasia mutated (ATM) mutation treated with definitive radiotherapy to metastatic sites, resulting in durable disease control at those sites and delay in needing systemic therapy.

## Case presentation

A 42-year-old man presented with right flank pain and gross hematuria. Abdominopelvic CT revealed a 6.0 cm right renal mass with a tumor thrombus extending into the infradiaphragmatic inferior vena cava (IVC). CT chest was negative for metastasis. Biopsy of the renal mass demonstrated a WHO grade 3 pRCC, favoring type 2 disease, with immunohistochemistry (IHC) positive for vimentin, CK20, CK7, and AE1/3. He underwent a right radical nephrectomy, with pathology showing a pT3b, WHO grade 3, renal cell carcinoma with mixed papillary and clear cell features as well as tumor extension into the renal sinus and IVC. IHC was positive for pankeratin, PAX8, CA-IX, EMA, vimentin, and AMACR found in RCC. Tumor cells were positive for TFE3 on IHC and while the fluorescence in situ hybridization (FISH) breakapart assay was negative for the TFE3 translocation, morphologic and IHC profiles were consistent with a MiT family translocation RCC. A Tempus xT 648-gene sequencing panel revealed somatic mutations, including ATM (c.4611+1G>a) splice region variant loss of function (LOF) and NSD1 (p.N272fs) LOF. Additionally, a germline BAP1 mutation was noted, indicative of a predisposition to BAP1 tumor syndrome.

The patient was discovered to have new precaval lymphadenopathy measuring up to 2.7 cm on surveillance imaging performed nine months post-nephrectomy, which was confirmed as metastatic RCC on biopsy. He initiated combination therapy with ipilimumab 1 mg/kg and nivolumab 3 mg/kg, receiving three cycles, with subsequent radiographic progression of previous lymphadenopathy and a newly enlarged left periaortic lymph node. He also developed severe symptomatic thyroiditis, followed by hypothyroidism during IO therapy. He was then transitioned to cabozatinib 40 mg daily but experienced multiple toxicities, including excessive fatigue, dysgeusia, mouth sores, nausea, and anorexia, which prompted multiple dose reductions. He was also hospitalized with fevers and hallucinations, which were ultimately deemed to be due to uncontrolled hypothyroidism. Restaging CT imaging three months post-cabozantinib, initiation showed newly enlarged left cervical lymph lymphadenopathy, which was biopsied and confirmed to be metastatic RCC. He was subsequently referred to radiation oncology due to poor tolerance of systemic therapy, limited availability of other systemic treatment options, and lymph-node-confined oligometastatic disease.

**Figure 1:**
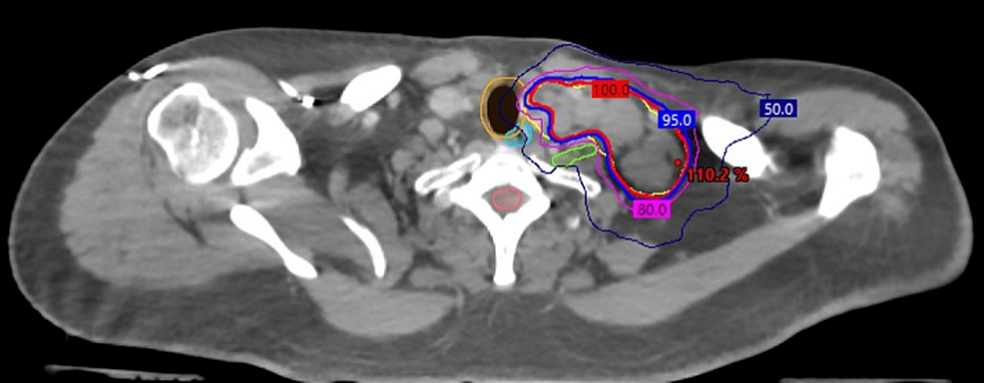
Radiotherapy treatment plans for oligometastatic papillary renal cell carcinoma (pRCC) of the left lower cervical lymph nodes Radiotherapy treatment planning computed tomography (CT) axial images are displayed for the management of pRCC involving the left lower cervical lymph nodes. The prescription dose is represented by the 100% isodose line (red) to a total of 60 Gy in 15 fractions. Regional hotspots are noted as point doses (red) with additional isodose lines of 95% (blue), 80% (pink), and 50% (navy). Nearby organs at risk (OARs) are contoured for (A): trachea (orange), esophagus (cyan), left brachial plexus (lime), spinal cord (salmon), and (B): liver (orange), duodenum (cyan), large bowel (brown), left kidney (yellow), spinal cord (burgundy).

**Figure 2:**
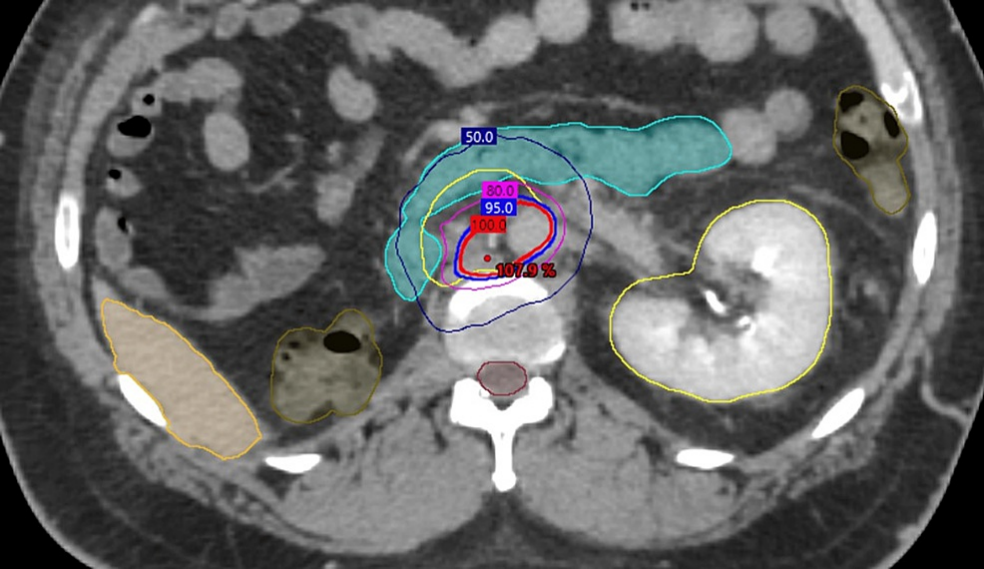
Radiotherapy treatment plans for oligometastatic papillary renal cell carcinoma (pRCC) of the retroperitoneal lymph nodes Radiotherapy treatment planning computed tomography (CT) axial images are displayed for the management of pRCC involving the retroperitoneal nodes. The prescription dose is represented by the 100% isodose line (red) to a total of 60 Gy in 15 fractions. Regional hotspots are noted as point doses (red) with additional isodose lines of 95% (blue), 80% (pink), and 50% (navy). Nearby organs at risk (OARs) are contoured for (A): trachea (orange), esophagus (cyan), left brachial plexus (lime), spinal cord (salmon), and (B): liver (orange), duodenum (cyan), large bowel (brown), left kidney (yellow), spinal cord (burgundy).

He was then planned for an aggressive course of radiotherapy, receiving 60 Gy in 15 fractions, to his cervical and retroperitoneal lymph nodes (Figures 1-2).

Both sites were treated utilizing volumetric arc therapy. For retroperitoneal lymphadenopathy, a 4D-CT to account for lymph nodes and organ at risk (OAR) motion was obtained. The duodenum was contoured on all breathing phases to generate internal OARs. An abdominal Vac-Lok and a long neck mask were utilized for immobilization. Dose constraints modeled after a recent trial examining radiation therapy in lieu of systemic therapy for oligometastatic RCC was used as well as a maximum point dose (D0.03 cc) of 45 Gy, which limited dose coverage of the retroperitoneal disease [9]. Due to significant nausea, vomiting, and weight loss, the patient only received 9 of the planned 15 radiation treatments to a total of 36 Gy, despite a 1-week treatment break between the fourth and fifth sessions. However, he was able to complete the entire course for his cervical lymphadenopathy to 60 Gy without any issues. One month following the completion of radiation, he developed acute radiation enteritis, which was treated with a one-month prednisone taper.

**Figure 3:**
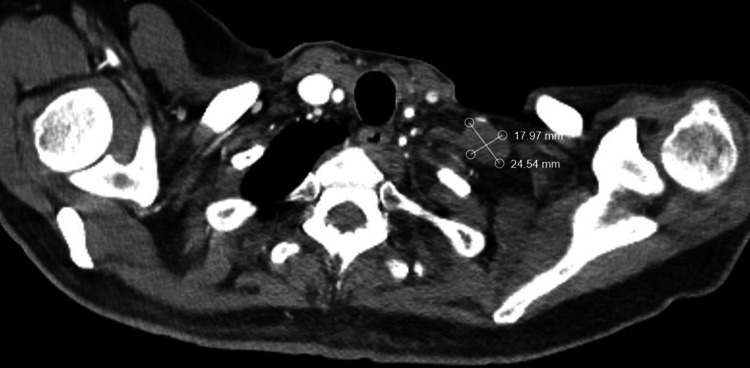
Latest surveillance axial computed tomography (CT) images of the chest Axial CT imaging scans with IV contrast re-demonstrating a stable left lower cervical 15 months after completion of radiation therapy. Measurements of residual disease demonstrate no significant progression by RECIST v1.1 criteria. RECIST: Response Evaluation Criteria in Solid Tumours

**Figure 4:**
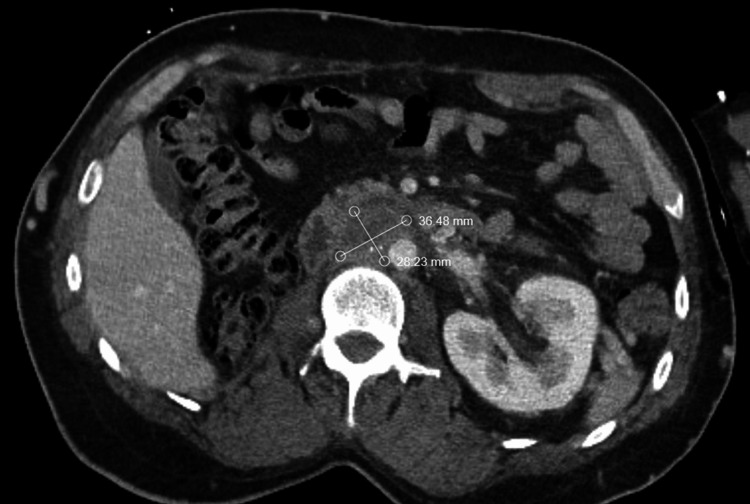
Latest surveillance axial computed tomography (CT) images of the abdomen and pelvis Axial CT imaging scans with IV contrast re-demonstrating retroperitoneal lymph nodes (B) 15 months after completion of radiation therapy. Measurements of residual disease demonstrate no significant progression by RECIST v1.1 criteria. RECIST: Response Evaluation Criteria in Solid Tumours

He was then trialed on olaparib 300 mg BID due to the presence of an ATM mutation on genomic testing but discontinued it one month later due to excessive fatigue, nausea, vomiting, and anorexia. He then received one cycle of pembrolizumab 200 mg, which was then discontinued due to similar gastrointestinal toxicity. He subsequently proceeded with a treatment break. Since that time, he has not developed any evidence of radiographic disease progression by RECIST (Response Evaluation Criteria in Solid Tumours) v1.1 criteria. He continues to have stable lymphadenopathy in the irradiated sites 15 months post-completion of radiation therapy as well as 3 years and 2 months post nephrectomy (Figures 3-4).

**Figure 5:**
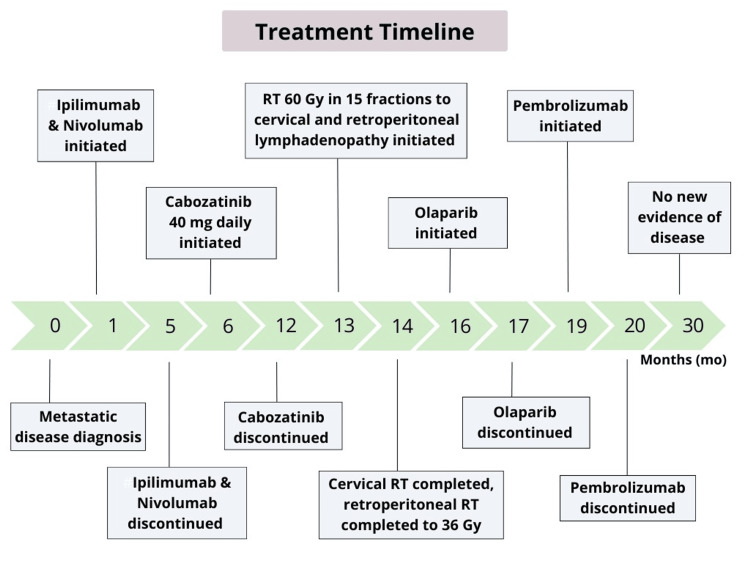
Patient’s treatment timeline An outline of the patient's treatment timeline starting at the time of diagnosis and detailing all of his systemic and radiation treatments until his last follow-up.

Figure 5 outlines the patient's entire treatment course.

## Discussion

In this report, our patient with metastatic papillary RCC (pRCC) remains alive and without disease progression 10 months after completing radiation therapy at the metastatic sites. Considering that PFS after first-line systemic therapy for pRCC is typically less than one year, this patient’s stable disease off all treatment is unexpected and prompts speculation into its cause [13-16]. Radiation therapy to his lymph nodes could explain this patient’s prolonged interval of stable disease. Radiation therapy for metastatic RCC to prolong PFS has been examined in prospective studies. For instance, a phase 2 trial from the University of Texas Southwestern (UTSW) examined stereotactic body radiation therapy (SBRT) to 57 metastases in 23 treatment-naïve patients with International Metastatic RCC Database Consortium (IMDC) favorable or intermediate risk mRCC and three or fewer sites of disease [10]. With a median follow-up of 21.7 months, freedom from systemic therapy and PFS at 1 year were 91.3% and 82.6%, respectively. MD Anderson (MDA) conducted a similar phase 2 study, except for allowing patients who failed one line of systemic therapy and had up to five sites of disease [9]. Additionally, this trial permitted prolonged courses of radiation beyond 5 fractions to allow for the treatment of metastases adjacent to more radiosensitive organs. At 1 year, the PFS was 64%. These trials and many other retrospective studies support that radiotherapy alone can prolong PFS for oligometastatic mRCC [6,7].

However, only 8.7% of patients enrolled in the UTSW trial [8] had pRCC histology, while the MDA study excluded non-clear cell RCC patients [7]. When compared to metastatic ccRCC, metastatic pRCC demonstrates lower median overall survival [17]. However, certain genomic characteristics may alter the expected prognosis and responses to therapies in pRCC. Our patient’s tumor contained a LOF ATM mutation, which occurs in only 3% of pRCC [18]. ATM is a key protein involved in double-stranded DNA break repair from ionizing radiation [19]. Multiple studies examining the effect of radiation or chemotherapy on tumors with impaired DNA repair ability have noted higher rates of response compared to their non-mutated counterparts [11,20]. As such, this patient’s pRCC may have been rendered more radiosensitive, which may explain his unusual treatment response despite receiving a suboptimal radiation dose to his retroperitoneal lymphadenopathy. Since the effects of DNA damage from radiation therapy can persist for months after completing radiation, it is feasible that the action of olaparib, a PARP inhibitor involved in DNA base repair [19], which our patient received within two months of finishing radiation therapy was augmented by the radiation. Conversely, the effects of radiation therapy may have been longer lasting due to olaparib therapy. The combination of PARP inhibition and radiation has been shown to be safe in early-phase trials, although its synergy remains poorly elucidated [21,22].

Five months after finishing radiation therapy, our patient also received a cycle of pembrolizumab. Typically, this interval is too long to be considered concurrent therapy. However, if the effects and duration of radiation were enhanced by olaparib, perhaps there was also increased expression of radiation-induced neoantigens to augment the action of pembrolizumab. In one study examining protein immunofluorescence following the administration of 15 Gy of radiation in 1 fraction one month before nephrectomy [23], higher levels of calreticulin were appreciated in the irradiated tumors compared to their non-irradiated cohort. Cell surface translocation of calreticulin is a molecular signal needed to achieve immunogenic cell death [24].

## Conclusions

In conclusion, we report a case of ATM-mutation metastatic pRCC with durable disease stability after receiving radiation therapy to the metastatic sites as well as a brief course of a PARP inhibitor and IO. We believe that this prolonged PFS is likely due to enhanced sensitivity of the tumor to radiation therapy due to ATM loss as well as augmented activity from the PARP inhibitor and IO therapy. This creates a potential opportunity for synergy between these therapies in pRCC, a rare subtype of renal tumors with limited standard treatment options.
